# The influence of household structure and composition on the introduction of solid, semisolid and soft foods among children aged 6–8 months: An analysis based on Ethiopia Demographic and Health Surveys

**DOI:** 10.1111/mcn.13429

**Published:** 2022-09-23

**Authors:** Asnake Ararsa Irenso, Miaobing Zheng, Karen J. Campbell, Dan Chamberlain, Rachel Laws

**Affiliations:** ^1^ School of Public Health Ambo University Ambo Ethiopia; ^2^ School of Exercise and Nutrition Science, Institute for Physical Activity and Nutrition (IPAN) Deakin University Burwood Victoria Australia; ^3^ Centre for Social Impact UNSW University of New South Wales Sydney New South Wales Australia

**Keywords:** Ethiopia, household composition, households, household structure, introduction of Solid, semisolid and soft foods, social network analysis

## Abstract

The early and late introduction of complementary food, both prevalent in Ethiopia, are associated with morbidities, growth faltering and developmental risks in children. The interhousehold network around the primary caregiver's intrahousehold network is critical in influencing the age of introducing complementary foods. This study examined the influence of household composition and structures on complementary food introduction. This is a secondary data analysis of four Ethiopian Demographic and Health Surveys conducted between 2000 and 2016. The household structure and composition variables were calculated from household members' kinship status and attribute, respectively. The introduction of solid, semisolid or soft foods was dichotomised as whether the children within 6 to 8 months have been given complementary foods. Multivariable logistic regression with adjustment for the primary caregiver and household characteristics was run to examine the associations between household structure and composition variables and the introduction of complementary foods. The marginal effects (ME) were calculated to facilitate the practical interpretation of the study findings. Large households (>3 nonredundant contacts) with extended family or unrelated people (high effective size, ME = 6.01%, 95% confidence interval [CI]: −8.53, −3.49) lowered the proportion of children starting food within the recommended 6–8 months. Households with close kins (high constraint) (ME = 7.22%, 95% CI: −13.65, 28.09) and greater age diversity (ME = 0.65%, 95% CI: 0.15, 1.15) increased the proportion of children receiving complementary food at an appropriate age. This study revealed that interhousehold structure and composition influence the age of introduction of complementary foods. These factors, therefore, need to be considered in designing interventions to improve age at the introduction of complementary foods.

## Key messages

- In a large family, women with extended and nonrelated household members had a 6% reduction in the proportion of children starting complementary food within 6–8 months.
- To our best knowledge, the negative influence of unrelated household members has not been reported before. Future studies might examine their roles and how this household subpopulation can be enabled to support optimal infant feeding.
- The higher constraint emanating from the interdependence of close kins as in a nuclear family or small household with extended members was associated with a 7.2% increment in the proportion of children starting complementary food at an appropriate age.

## 1 INTRODUCTION

Multiple burdens of malnutrition, including undernutrition, overnutrition and poverty, have become integral problems in low‐income countries (Haddad et al., 2015). In Ethiopia, stunting driven by suboptimal feeding practices, among other causes, affects more than two of five children, and undernutrition accounts for 28% of child deaths (FDRE, 2013). One of these feeding practices against the recommendation is the age of introduction of complementary foods, measured by the proportion of children 6–8 months given solid, semisolid and soft foods in the last 24 h (WHO, 2008).

At 6 months of age, breast milk alone can no longer meet child nutritional requirements, and early and late introduction of solid food carries growth and development risks (WHO, 2008). However, the proportion of Ethiopian children starting complementary food within the appropriate age range (6–8 months) remains a problem, with a pooled national prevalence of 62.5%, which increases with women giving birth at health care institutions and lower among working women (Habtewold et al., 2019; Yeheyis et al., 2016).

The prevalence of timely introduction of solid food varies according to geographical location, with 53% to 72% of infants living in predominantly rural areas introducing solid foods in a timely way (Anteneh et al., 2017; Biks et al., 2018; Kassa et al., 2016; Kim et al., 2016) compared to 55.2% to 83% in the capital. This is largely attributed to the higher educational status of those living in urban areas (Mohammed et al., 2018; Yeheyis et al., 2016). Complementary foods are delayed among food insecure rural Ethiopia households due to knowledge gaps on age‐appropriate complementary feeding practices (Hirvonen et al., 2021). While there is no recommendation, according to the United Nations Office for the Coordination of Humanitarian Affairs (OCHA), less than 80% of children meeting this criterion are considered a public health priority (OCHA, 2021).

In Ethiopia, the typical nonbreast milk liquids and solids given before 6 months include water and cow, goat or camel milk, butter, yogurt and injera—a solid food based on local staples (Bililign et al., 2016; Mekonnen et al., 2018; West, 2017). Early introduction of these foods increases the risk of pneumonia (Nirmolia et al., 2018), diarrhoea (Anteneh et al., 2017; Asfaha et al., 2018; Ogbo et al., 2016, 2017, 2018), anaemia (Gebreweld et al., 2019), and severe acute malnutrition (Pravana et al., 2017; Tufa et al., 2018). The frequently delayed solid foods are meat, fruit, and vegetables, which decreases dietary diversity and raises the risk of multiple concurrent nutrient deficiencies (Dhami et al., 2019; Kim et al., 2016; West, 2017).

Averting undernutrition and enhancing children's survival requires focusing on multiple interventions within enabling environments, including the household (Black et al., 2020). The household is the most proximal setting where thriving can be promoted by delivering responsive care, safe and nutritious foods, health care, learning opportunities, safety and security (Black et al., 2020).

In a broader sense, the pathway from the household composition and structure to optimal nutrition is mediated by adults' physical presence and attention to the child. Adults can be missing from the family due to marital dissolution, working away from home (Coleman, 1988), underreported orphanhood during early life (Robertson et al., 2008) from HIV/AIDS (Kebede et al., 2000; Mirkuzie et al., 2020), the high maternal mortality rate (Tessema et al., 2017) or other causes (UNICEF, 2022). The absence of adults leads to an environment where the child's nutrition is at risk (MacDonald et al., 2020).

The mothers/primary caregivers are the ultimate decision‐makers for infant feeding among household members (Benoit, 2004). However, maternal decisions occur within a household context where other actors may exert their influence (Dewey, 2003). Thus, it can be theorised that while a mother's physical presence is essential to promoting an infant's optimal nutrition, it may not be sufficient, as infant feeding may be hampered or facilitated by others within the network (Abebe et al., 2019; Coleman, 1988; Federal Ministry of Health, 2004; Lucas et al., 2018).

Thus, the role of the mother's intrahousehold social network is critical around the age of 6 months when complementary foods must be started (WHO, 2008). The timing of introduction might be guided by social norms related to parenting practices, gender, local culture, the types of support the mother/caregiver obtains and household members' roles (Bililign et al., 2016; Kinabo et al., 2017; Mekonnen et al., 2018; Reda et al., 2019; Tamiru et al., 2013). The cumulative effect of these factors on the timing of the introduction of solid foods shapes the diet composition, patterns, current and future food preferences, habits and wellbeing of the child (Adair, 2012; Owino, 2019).

Household members also undertake diverse child feeding roles based on their age, sex and kinship status. For instance, support obtained from fathers' (Reda et al., 2019) improves the timely initiation of complementary foods in Ethiopia (Biks et al., 2018; Kim et al., 2016). Further, in a study conducted in Southern Ethiopia involving 764 caregivers in 2009, four out of five caregivers were biological mothers, and they preferred using a responsive feeding style (Wondafrash et al., 2012). In contrast, nonbiological caregivers practised laissez‐faire feeding styles, characterised by minimal child encouragement to eat, irregular child feeding patterns and timing of feeding (Dettwyler, 1989; Wondafrash et al., 2012).

Overall, early or late complementary feeding is an individual decision (Underwood & Hofvander, 1982) driven by social factors and opportunity rather than meeting the child's nutritional requirements (Przyrembel, 2012). Addressing social factors in infant feeding practices is a high priority for the Ethiopian Government. The National Infant and Young Child Feeding Strategy recognises the need to focus beyond the mother–child dyad to involve fathers and other household members via companionship, access to accurate information and caring (Federal Ministry of Health, 2004; Przyrembel, 2012). This focus builds on research that shows that improving fathers' (Mukuria et al., 2016) and grandmothers' knowledge (Karmacharya et al., 2017; Mukuria et al., 2016) of infant feeding translates to better infant feeding practices by the mother.

Previous Ethiopian Demographic and Health Survey (EDHS) analysis and other studies on determinants of the introduction of complementary feeding have focused on individual‐level factors, including maternal, child, paternal or other family members or household attributes (Ahmed et al., 2020; Przyrembel, 2012). Studies have not explored the introduction of complementary foods from the broader household environment, including the parent–child dyad and the composition and structure of households. This information is important in understanding persistent suboptimal practices and will inform opportunities to support healthy child development within Ethiopian households. Hence, this study will provide an in‐depth analysis of the influence of household composition and structures and associations with the introduction of solid, semisolid or soft foods between ages 6 and 8 months.

## 2 METHODS

### 2.1 Study design and participants

This study is a secondary data analysis based on the four EDHS rounds conducted since 2000. The EDHS used a two‐stage cluster design, where geographic areas were sampled first, and households comprised the second sampling stage (ICF International, 2012). The EDHS was conducted in nine geographical regions and two city administrations, thus nationally representative. Regions and city administrations vary in size, with the risk of oversampling for small and undersampling for small and large populations, respectively. Thus, representativeness at the national and subnational levels is ensured by applying a mathematical adjustment, the sampling weight for women and children (ICF International, 2012).

The EDHS conducted every 5 years, with varying study periods (The DHS Program & ICF, 2019): EDHS 2000 (February 2000–May 2000) (CSA & ORC Macro, 2000), EDHS 2005 (April 2005–August 2005) (CSA & ORC Macro, 2006), EDHS 2011 (December 2010–June 2011) (CSA & CF International, 2012) and EDHS 2016 (January 2016–June 2016) (CSA & ICF, 2016). The current analysis utilised household data, which lists all the usual members and visitors and their identifying information, with children between 6 and 8 months of age whose caregivers responded to the infant feeding practice questions from the household questionnaire (Rutstein & Rojas, 2006).

The data were collected following appropriate ethical procedures, available on the four rounds of EDHS reports for EDHS 2000 (CSA & ORC Macro, 2000), 2005 (CSA & ORC Macro, 2006), 2011 (CSA & CF International, 2012) and 2016 (CSA & ICF, 2016). The DHS Program approved the data access on April 13, 2020.

### 2.2 Variables

Variable selection was based on a conceptual framework of determinants of feeding practices for children older than 6 months developed by Hector et al. (2005) as cited by Blaney et al. (2014, 2015). The outcome variable is the introduction of solid, semisolid or soft foods and exposure variables related to household composition and structure variables as described below.

#### 2.2.1 Introduction of solid, semisolid or soft foods

This is one of the core indicators for infant feeding practices, defined as the proportion of infants 6–8 months who received solid, semisolid or soft foods. The indicator value was derived from questions administered to the child's primary caregiver after obtaining voluntary informed consent (The DHS Program & ICF, 2019), asking them to recall the child's dietary intake on the previous day (with No/Yes response), and verifying their response by asking what kind of solid, semisolid or soft foods did the child eat. This variable definition and measurements are available in the updated WHO/UNICEF document (WHO & UNICEF, 2021). The outcome variable was analysed as a binary variable: whether or not to receive solid foods at 6–8 months of age.

#### 2.2.2 Women's intrahousehold network measures

The analysis focused on the social network around the women respondent, the primary caregiver (the mother or other women in the household) who responded to the infant and young child feeding questionnaires. The EDHS household roster used to collect the data contains 20 lines, including the women respondents and their demographic information, including age, sex, education and relationship to the household's head and coresidence status. These variables were used to calculate the composition and structure variables. In this analysis, women respondents are referred to as egos. Any other individuals listed on the household roster are referred to as an altar, and the relationship among them, alter–alter tie. The social network measures were derived using E‐Net version 0.050 (Borgatti, 2006) and imported to Stata 15 (StataCorp, 2013) for further analysis.

#### 2.2.3 Household composition measures

The index of qualitative variation (IQV) was calculated to estimate the alters’ heterogeneity based on categorical variables, such as people with diverse relationships, usual household residents (de jure members), actual household residents (defacto members), sex and educational status. E‐Net software provides two summary diversity measures, Blau index (H) and IQV (Borgatti, 2006). Suppose the variable has *r* number of different categories and *P_i_* is the proportion of categories in variable *i*, then the Blau index *H* is:

$$H=1-{P_{1}}^{2}-{P_{2}}^{2}-{P_{3}}^{2}-\text{…}\text{…}.-{P_{r}}^{2}.$$

The IQV is a normalised version of the Blau index weighted for the number of categories, calculated as follows (Crossley, 2019):

IQV=H1−1r.

De facto members are people who actually slept in the household the night before the data collection, including visitors. Most household members are both de jure and de facto, that is, those usual members who stayed in the household the night before the survey. However, some are de facto but non de jure (e.g., Visitors), which gives IQV of the de jure figure; a visitor means diversity to usual household members. Other household members are de jure but nondefacto, that is, a usual household member staying away from home gives diversity to those staying at the household, the IQV of de facto members.

The IQV Ranges from 0 to 1, with higher scores, indicating more heterogeneity (0 = all alters in the same category; 1 = alters equally dispersed across all categories) (Solanas et al., 2012). For educational status, usual household members and actual household members, the IQV was dichotomised based on the median value and coded as low diversity for values below the median and high diversity for values above the median value. The alters' age diversity was measured on a continuous scale and estimated using standard deviation (SD). The detailed analysis plan has been previously published (Irenso et al., 2021).

#### 2.2.4 Household structure

The household structure was constructed using ties among alters, who are listed as household members with women (alter–alter tie). The alter–alter ties were created based on the kinship status of household members estimated with the coefficient of relatedness, tie present if each pair of alters were related, otherwise absent guided with the value of 0.5 (for parents and children); 0.25 for grandparent and grandchild; 0.125 for nephew or nieces, and zero (missing ties) for any combination of nonrelated household members (Koyama, 2016).

The alter–alter ties helped to calculate the structure variables, including degree, effective size and constraint, measured on a continuous scale (Labun & Wittek, 2016). The degree is the number of people in the women's household (number of alters), excluding her. Effective size is the number of nonredundant contacts, and it is the alternative way of measuring the network size after controlling for similar ties/redundancies (relationship among alters) that each alters has to the other, a measure defined based on connections. Effective size ranges from 1 (a woman with one alter or all alters are related) to *N* (all contacts are unrelated to each other), with *N* the degree(Labun & Wittek, 2016). A woman with a larger effective size is associated with diversified ties. The effective size was dichotomised based on the median as small ≤3 and high >3 effective sizes for analysis.

Constraint describes the extent to which the primary caregivers have access to related household members (Everett & Borgatti, 2020). This can have positive and negative influences, depending on the support mechanism. Suppose the intrahousehold network mechanism is interdependence, such as sharing the women's time. constraints; in that case, the high degree of trust and reciprocity and a shared sense of responsibility to caregiving might positively influence the introduction of complementary foods.

Alternatively, the constraint mechanism can be based on the structural hole argument, that is, with the presence of people who have a weak link to the household members, such as the visitors or nonrelated, have potentially different views and experiences from the rest of the household members. Thus, their suggestions to the women might differ from other households and lead to new information and emotional or in‐kind support. Compared to closely related members with redundant caregiving resources, people with weak ties to the household bring different perspectives to primary caregivers, that is, more behavioural constraints with the former than the latter (Everett & Borgatti, 2020; Lin et al., 2001). The constraint score ranges from 0 to 1 (0–100 percentage points). The higher the number of women contacts, the lower the constraints; when all alters are unrelated (no tie among alters), the score is 0. When alters are nuclear family members, the constraint increases to 1. The lower the constraint score, the lower the constrained behaviour (Labun & Wittek, 2016).

#### 2.2.5 Covariates

Covariates include the respondent's (all are women) characteristics (Chen, 2013), such as residence (urban/rural), educational status, age, occupation, sex of households' head and overall household characteristics of socioeconomic status, the wealth index.

### 2.3 Statistical analysis

All analyses were conducted in STATA 15 with statistical significance set at *p* < 0.05. Sampling weight was considered in all analyses using ‘svy’ command. The introduction of solid, semisolid or soft foods and other categorical variables were described using percentages. Continuous variables were described using mean and SD or the median with 25th and 75th percentile.

Multivariable logistic regression was conducted to examine the associations between household structural and compositional variables and the introduction of complementary foods at 6–8 months of age. Analyses were initially conducted separately in each survey round. As the household structural and compositional variables were highly correlated, each structural and compositional variable was analysed in separate logistic regression models. The crude model included each household structural and compositional variable as the exposure and the introduction of solids, semisolid or soft foods as the outcome. The covariates were women's characteristics (i.e., age in years, educational status, residence, coresidence with husband/partner, sex of household head, wealth index and type of earnings to obtain an overall adjusted odds ratio). The overall pooled effects across all survey rounds were also assessed using multilevel mixed‐effects logistic regression models specifying the survey year as a random effect, assuming all people in the household have the same weight.

The pooled associations from the multilevel mixed‐effects regression model were converted to the marginal effects (ME) to facilitate the practical interpretation of the study findings. The ME was interpreted as an incremental change in the percentage of children who started solid foods at 6–8 months (multiplied by 100 and reported as a percentage point change/actual amount of change) associated with a unit increase in value for continuous explanatory variables. For categorical variables, the ME was interpreted as the mean percentage point difference in the proportion of children given complementary foods at 6–8 months between the category of interest and the reference category (Norton et al., 2019).

### 2.4 Ethical statement

Deakin University Human Research Ethics Committee (DUHREC) waived ethical review with approval number 2020‐279 on August 20, 2020. The waiver was provided as per the National Statement on Ethical Conduct in Human Research (2007, updated 2018) Section 5.1.22 as it meets the definition for negligible risk research (as defined in National aph 2.1.7) and involves only the use of pre‐existing collections of nonidentifiable data about human beings.

## 3 RESULTS

Children aged between 6 and 8 months (*n* = 1744) and their household members with complete pertinent data were included in the analysis. Disaggregating over survey rounds, the number of unweighted samples for EDHS 2000, 2005, 2011 and 2016 was 458, 224, 551 and 511, respectively.

The mean age of the women respondents was comparable across the survey rounds, ranging from 27.9 to 28.9 years. The proportion of women with primary school level education and above spanned from 11.2% (EDHS 2000) to 34.0% (EDHS 2005), with most women having no formal education across all survey rounds. The proportion of female household heads increased from 5.3% in 2000to 14.1% in 2016. For the type of earnings, apart from EDHS 2000, where more than a third were not working, more than half of women were not working during subsequent survey rounds (Table 1).

**Table 1:** Participant characteristics of households with children 6–9 months EDHS 2005–2016 (weighted)

Variables	2000 (*n* = 542)	EDHS 2005 (*n* = 256)	2011 (*n* = 579)	2016 (*n* = 529)
	*n* (%)	*n* (%)	*n* (%)	*n* (%)
Women characteristics				
Women age in years (mean ± SE)	28.4 (0.47)	27.9 (0.53)	28.9 (0.40)	28.0 (0.39)
Educational status				
No formal education	481 (88.8)	169 (66.2)	429 (74.1)	363 (68.6)
Primary school and above	61 (11.2)	86 (33.8)	150 (25.9)	166 (31.4)
Residence				
Urban	41 (7.5)	75 (29.4)	73 (12.6)	101 (19.1)
Rural	501 (92.5)	181 (70.7)	506 (87.4)	428 (80.9)
Sex of household head				
Female	29 (5.3)	24 (9.9)	57 (9.7)	75 (14.1)
Male	513 (94.7)	232 (90.1)	523 (90.1)	454 (85.9)
Type of earnings				
Not working	186 (34.3)	167 (65.1)	366 (63.2)	274 (51.8)
Working but not paid	145 (26.8)	17 (6.8)	92 (17.5)	92 (17.5)
Paid in cash In‐kind	211 (38.9)	72 (28.1)	162 (23.9)	162 (30.7)
Household structure				
Degree (median; Q1, Q3)	5 (3.0,6.0)	5 (3.0,6.0)	5 (3.0,6.0)	5 (3.0,7.0)
Effective size (median; Q1, Q3)	3 (2.2, 3.8)	3 (2.0, 4.0)	3 (2.3, 4.0)	3 (2.0, 4.1)
Constraints (median; Q1, Q3)	0.56 (0.44, 0.64)	0.56 (0.44, 0.75)	0.56 (0.44, 0.64)	0.53 (0.44, 0.65)
Wealth index				
Poor	194 (35.7)	55 (21.6)	205 (35.4)	168 (31.9)
Middle	228 (42.1)	57.7 (22.6)	196 (34.0)	178 (33.6)
Rich	120 (22.2)	143 (55.8)	177 (30.6)	183 (34.5)
Household composition				
Coresidence with husband/partner				
Living with women	517 (95.5)	235 (91.8)	540 (93.3)	453 (85.6)
Stay elsewhere	24 (4.5)	21 (8.25)	39 (6.73)	76 (14.4)
Types of the respondent				
Nonmaternal caregivers	40 (7.3)	29 (11.3)	27 (4.6)	74 (13.9)
Maternal caregivers	502 (92.7)	227 (88.7)	552 (95.4)	455 (86.1)
SD of age in the year (median; Q1, Q3)	13.8 (11.9, 16.6)	13.6 (11.5, 16.3)	13.3 (11.6, 16.0)	13.1 (11.0, 15.5)
IQV of sex (median; Q1, Q3)	0.89 (0.75, 0.98)	0.89 (0.75, 0.96)	0.89 (0.64, 0.96)	0.89 (0.75, 0.98)
IQV of kinship types	0.75 (0.64, 0.94)	0.89 (0.64, 0.94)	0.75 (0.56, 0.89)	0.75 (0.56, 0.92)
IQV of educational status (median split)				
Low diversity	412 (76.1)	156 (60.9)	290 (50.4)	266 (29.1)
High diversity	129(23.9)	100 (39.1)	286 (49.6)	779 (40.9)
IQV de facto household members (at least one usual member who has not slept in the house)				
Diversity absent (ref)	465 (85.9)	206 (80.5)	504 (87.4)	445 (84.1)
Diversity present	77 (14.1)	50 (19.5)	73 (12.6)	84 (15.9)
IQV de jure household members (The presence of at least one visitor)				
Diversity absent (ref)	470 (86.8)	232 (90.5)	543 (94.1)	449 (84.8)
Diversity present	72 (13.3)	24 (9.5)	34 (5.92)	80 (15.2)
Child introduced to solid, semisolid and soft foods				
No	350 (64.5)	120 (47.0)	295 (51.0)	211 (39.9)
Yes	192 (35.5)	136 (53.5)	284 (49.0)	318 (60.1)

Abbreviation: IQV, index of qualitative variation.

For household structural variables, the median number of people living with the women (degree) was five, with an interquartile range (IQR) of three to four across survey rounds. After controlling for similarity of kinship categories (effective size), the median number of people living with women was three. The median constraints across survey rounds were 0.56, indicating that 56% of alters were related at the median, with higher variability of constraints in 2005 and 2016 at IQR of 33% and 21%, respectively.

Among household compositional variables, the proportion of husbands staying away from the household increased from 4.5% in 2000to 14.4% in 2016. For the type of survey respondent, the proportion of nonmaternal caregivers increased from 7.3% in 2000 to 13.9% in 2016. The IQV of sex (the sex diversity) was similar across EDHS rounds at the median of 89%, with no marked variability. The diversity of kinship types among alters was higher at the median value of 89% in 2005 than 75% in the other rounds, IQR ranging from 30% to 36%. Alters had low educational diversity in the 1st two rounds of the survey, where most had no formal or primary education compared to the latter two rounds, with more even distribution across all educational statuses, including secondary and higher education. The IQV of de facto household members, at least one usual household member staying away from home, ranged from 12.6% in 2011 to 19.5% in 2005. The IQV of de jure household members, at least one visitor, ranged from 5.9% in 2011 to 15.2% in 2016. During the survey, the proportion of children introduced to solid, semisolid and soft foods between 6 and 8 months has shown improvement from 35.5% in 2000 to 60.1% in 2016.

### 3.1 Factors associated with introduction to solid, semisolid or soft foods

Overall, the effective size (the number of alters adjusted for similar kinship categories) significantly decreased the odds of introducing solid semisolid and soft foods at 6–8 months (Table 2). The more alters kinships types are nonredundant, and the effective size of >3, decreased the proportion of children introduced to complementary foods by 6.01% (95% confidence interval [CI]: −8.53%, −3.49%) compared to those with three or fewer kinship categories. An inverse relationship between effect size and the introduction of solids was found throughout the four survey rounds. However, constraints (the more related household members, hence, more dependence) tended to positively influence age at the introduction of complementary foods (ME = 7.22%, 95% CI: −13.65%, 28.09%), that is, the more related people the household have, the better. The association shifted from positive in 2000 to 2011 to negative in 2016. In contrast, no evidence of an association was found for the degree.

**Table 2:** Factors associated with child introduced to solid, semisolid and soft foods, EDHS 2000–2016

Variables		EDHS round								Overall AOR^c^	Marginal effect (%)^d^
		2000 (*n* = 542)		2005 (*n* = 256)		2011 (*n* = 579)		2016 (*n* = 529)			
		COR^a^ (CI)	AOR^b^ (CI)	COR (CI)	AOR (CI)	COR (CI)	AOR (CI)	COR	AOR		
Household structure											
Degree		0.94 (0.83, 1.06)	1.04 (0.88, 1.22)	0.83* (0.69, 0.99)	0.86 (0.69, 1.08)	0.96 (0.87, 1.07)	0.99 (0.86, 1.12)	1.04 (0.94, 1.15)	1.0 (0.90, 1.10)	0.98 (0.94, 1.02)	−0.51 (−1.39, 0.37)
Effective size											
Low effective size (ref)											
High effective size		0.62 (0.37, 1.05)	0.75 (0.41, 1.38)	0.56 (0.28, 1.12)	0.74 (0.33, 1.640)	0.74 (0.46, 1.18)	0.79 (0.45, 1.36)	1.15 (0.66, 1.99)	0.90 (0.45, 1.82)	0.78*** (0.7, 0.87)	−6.01*** (−8.53, −3.49)
Constraints		3.31 (0.78, 14.09)	1.21 (0.18, 7.94)	8.41 (0.99, 71.49)	4.39 (0.32, 60.68)	2.17 (0.52, 9.04)	1.56 (0.26, 9.37)	0.27 (0.07, 1.09)	0.36 (0.07, 1.90)	1.35 (0.56, 3.24)	7.22 (−13.65, 28.09)
Household composition											
Age diversity (SD of age in years)		1.01 (0.95, 1.06)	1.03 (0.96, 1.09)	1.02 (0.95, 1.08)	1.05 (0.97, 1.14)	1.01 (0.96, 1.05)	1.02 (0.96, 1.07)	1.05 (1.0, 1.10)	1.06* (1.01, 1.13)	1.03* (1.01, 1.05)	0.65* (0.15, 1.15)
IQV of sex		0.80 (0.35, 1.81)	0.73 (0.31, 1.70)	0.52 (0.18, 1.50)	0.69 (0.22, 2.16)	0.77 (0.38, 1.55)	0.84 (0.39, 1.78)	1.27 (0.52, 2.95)	1.14 (0.46, 2.83)	0.83 (0.68, 1.02)	−4.37 (−9.09, 0.36)
IQV of educational status											
Low (ref)											
High		1.28 (0.75, 2.20)	1.02 (0.57, 1.84)	1.28 (0.75, 2.2)	0.50 (0.21, 1.15)	1.10 (0.66, 1.83)	1.04 (0.58, 1.84)	1.32 (0.75, 2.32)	1.34 (0.71, 2.52)	1.07 (0.78, 1.47)	1.59 (−6.10, 9.28)
De facto family members											
Diversity absent (ref)											
Diversity present		0.95 (0.42, 2.13)	1.04 (0.47, 2.33)	1.02 (0.46, 2.260	1.32 (0.54, 3.23)	1.37 (0.65, 2.91)	1.4 (0.66, 3.00)	1.09 (0.53, 2.23)	1.13 (0.56, 2.32)	1.14 (0.94, 1.37)	3.08 (−1.50, 7.66)
De jure family members											
Diversity absent (ref)											
Diversity present		0.78 (0.35, 1.75)	0.74 (0.31, 1.77)	0.70 (0.22, 2.26)	1.14 (0.33, 3.97)	1.26 (0.52, 3.08)	0.92 (0.37, 2.31)	1.37 (0.66, 2.86)	1.49 (0.64, 3.46)	1.09 (0.80, 1.50)	2.09 (−5.50, 9.69)
IQV of kinship types		1.86 (0.55, 6.38)	0.44 (0.07, 2.87)	2.74 (0.45, 16.51)	5.05 (0.39, 65.80)	1.78 (0.64, 4.96)	2.11 (0.50, 8.99)	0.96 (0.32, 2.91)	2.87 (0.72, 11.46)	1.82 (0.83, 4.02)	14.35 (−4.99, 33.7)

Abbreviations: AOR, adjusted odds ratio; CI, confidence interval; COR, crude odds ratio; IQV, index of qualitative variation.

^a^ Crude model includes each family structure and composition variable as the exposure and introducing the child to solid semisolid and soft foods as the outcome using ‘svy’.

^b^ Adjusted model adjusted for covariates including women's age in years, educational status, residence, coresidence with husband/partner, sex of household head, wealth index and type of earnings using ‘svy’.

^c^ Overall OR was calculated with multilevel mixed‐effects logistic regression modelling specifying survey year as a random effect.

^d^ The average marginal effect was calculated for each variable as the discrete change from the reference value for categorical variables (effective size, de facto family members and de jure family members) and a probability of children introduced to solid, semisolid and soft foods with a small change for continuous variables from the multilevel mixed‐effects logistic regression model.

* *p* < 0.05;

*** *p* < 0.001.

A unit change in the alters age diversity was associated 0.65% increase in children introduced to solid, semisolid and soft foods (95% CI: 0.15%, 1.15%). Conversely, sex diversity (when all members are male or female vs. an equal distribution) seemed to decrease the proportion of children receiving timely introduction of complementary foods by −4.37% (95% CI: −9.09%, 0.36%). All other compositional variables, including IQV of educational status and higher diversity in de facto and de jure household members, tended to increase the proportion of children introduced to complementary foods on time. Similarly, except during the EDHS 2000, IQV of kinship types appeared to increase the proportion of children introduced to complementary foods on time by 14.35% (ME = 14.35%, 95% CI: −4.99%, 33.7%), that is, the even distribution of alters across kinship types, the better timing of solid food introduction—the association shifted from negative in EDHS 2000 to positive in later survey rounds, but not statistically significant.

## 4 DISCUSSION

This study aimed to assess the influence of household composition and structure on the introduction of solid, semisolid or soft foods for infants aged 6–8 months in Ethiopia. We found that caregivers with more than three nonredundant contacts, which are expected to include extended family members and unrelated people, significantly decreased the proportion of children starting complementary foods on time. Conversely, infants from households made up of closely related members of varying ages and educational statuses were more likely to be introduced to complementary foods on time.

This study shows improvements over time in the proportion of infants given solid, semisolid or soft foods between 6 and 8 months, from only one in three children in 2000 to three in five in 2016. While this is positive, two in five infants between 6 and 8 months still do not start complementary foods, which is against the WHO recommendation (WHO, 2008). Subsequently, when introduced late, this delay in consumption of complementary foods increases the risk of infant stunting (Dhami et al., 2019; Hirvonen et al., 2021), while early introduction increases the risk of being underweight and wasting (Masuke et al., 2021).

The low proportion of timely introduction of foods across survey rounds might relate to the cultural persistence of feeding traditions across generations. When deciding when to introduce the food, the primary caregiver is guided by her beliefs and external feedback—whether positive or negative, received implicitly or explicitly from her social network (Harrison et al., 2017; Worobey, 2016). The negative impact on the timely introduction of foods conferred by extended and nonrelated members in larger households highlights the complexity of women's decision‐making dynamics. In this regard, women from a small nuclear family household with or without extended members, which keeps the effective size close to one, have better outcomes. The smaller households have more educated members and are better off economically than larger ones. The advantage of a smaller family can be expanded by empowering the women and enhancing their bargaining power through education, family planning services and economic autonomy (Bhargava, 2007; Dibaba, 2009; Doepke & Tertilt, 2018).

However, the effective size surpasses three in large households with extended and nonrelated family members, resulting in the early or late introduction of complementary foods. The mechanism behind negative influence might be related to women's time poverty, where her time investment in the household and beyond (Irenso et al., 2022; Sik, 2020) coincides with disproportionately low support from others to feed her infant appropriately. The alternative explanation of the negative relationship might be related to the household decision‐making dynamics of homogamy where the child's parents are of similar background, including low economic and educational status, cultural background and parental attitude. These similarities may mean that these families are more likely to follow traditional practices that are contrary to optimal infant feeding. Thus, homogamy is common in Ethiopia and potentially perpetuates existing incorrect feeding practices (Kalmijn, 1998).

Our findings showed that nonrelative household members modify the household structure and negatively influence the timing of the introduction of complementary foods. Evidence shows that nonrelated people come to the household as domestic servants (Biadegilegn, 2011). In Ethiopia, domestic workers comprise 3.3% of all employment, higher for females at 5.3% than males at 1.6%. Most of these female domestic workers reside in their employer's household, undertaking household chores and child caregiving (ILO, 2022). Domestic workers, for instance, in Addis Ababa, are adolescents, migrate from rural to urban areas and are less educated (Erulkar & Ab Mekbib, 2007). These unrelated people are heavily involved in childcare. However, their involvement in child feeding activities and practices has never been assessed in Ethiopia. Based on these premises, domestic servants are a stealth subpopulation to be considered in infant feeding. Further research describing the impact of this group on child feeding is required.

Among household composition variables, women whose alters are of different ages (age diversity) and had a better chance of offering complementary food on time. Age is one of the social categorisation variables in determining group membership. In social categorisation, people close in age to the mothers might seek and share similar messages and act similarly, providing peer support in the household. With increasing adult age diversity, the presence of both old and young alters might limit the acceptance of suggestions of older household members (McNicoll, 2002), such as food distribution traditions that prioritise elders and husbands (Berhanu et al., 2019) and the early introduction of complementary food for boys but not girls (Andrysek et al., 1984). Evidence shows a growing sentiment among the older generation that their long‐held norms are undervalued (Adamek et al., 2019).

This study shows that in households with even distribution members across kinship types, the better timing of introducing complementary food, the association between IQV of kinship types and the introduction of solid, semisolid and soft foods from negative in 2000 to positive in later survey rounds. One possible explanation for this might be related to opinion diversity that converges to a more positive deviant behaviour, which corresponds to national policy such as the implementation of the Health Extensions Programme in 2004, which brought better access to health prevention services compared to the year 2000 (Banteyerga, 2011). As an alternative explanation, this study showed that women's educational status has improved over subsequent survey rounds, hence more empowerment to choose and make the right decision, even with conflicting messages from household members (Berhane et al., 2001). Hence, households of multiple generations are likely to be more supportive and empowered, and the role of household members, such as the senior members, deviates from positive behaviours.

Modifying existing practices requires understanding cultural community (Kalra et al., 2018). The introduction of complementary food provides an opportunity for early food acculturation, socialisation of the infant into the household and community foods. In the process of introducing the child to foods, other household members observe, learn and preserve the practices (Palmer, 2011). The Ethiopian food and nutrition policy recognises the cultural process and contextualises nutrition messages to promote optimal nutrition practices among families and communities (Federal Democratic Republic of Ethiopia, 2018). Indeed, previous studies showed that educational intervention improves the appropriate initiation of complementary feeding (Arikpo et al., 2018; Hirvonen et al., 2021), but the cultural aspect has not been well addressed. Thus, future intervention development might include a cultural evaluation, beginning with identifying shared beliefs, acknowledging variations across Ethiopia, framing the messages accordingly and implementing them across different sectors, including health and nonhealth sectors.

The national strategy for improving complementary foods also includes delivering appropriate messages to parents and other caregivers and removing cultural and practical barriers. Our findings showed barriers (e.g., nonrelatives and extended members in large households) and facilitators (e.g., nuclear family households) to translating intrahousehold networks around the mother by supporting her and improving infant feeding practices. For instance, supporting women's nutrition during pregnancy improves infant feeding (Dewey, 2001) by reducing the perception that the newborn is small (Issaka et al., 2014) and that the breast milk is insufficient for the baby, a common justification for the early introduction of food; these claims are reinforced by the grandmothers and other females in the household (Kalra et al., 2018). Further, economically disadvantaged caregivers (Hazir et al., 2012), women with less frequent antenatal care (Andrysek et al., 1984; Hazir et al., 2012) and fewer postnatal care visits (Yohannes et al., 2018) have an increased risk of early introduction of complementary foods. These factors call for stakeholders to examine the communication strategy to empower the household members to support the caregivers holistically.

Importantly, this study provides insights into the complex household factors influencing the introduction of solid, semisolid and soft foods using multiyear nationally representative data, with social network analysis methods at a household level. The findings of this study are a proxy for the embedded sociocultural context of infant feeding practices. The findings stimulate further in‐depth studies describing how unrelated people negatively influence infant feeding and testing what intervention approach works for large households with extended and unrelated members. While describing the complexity of households is a strength, it is acknowledged that households represent just one part of the complex social dynamics that are likely to influence child feeding. Future works might expand the social network analysis methods to examine the influence of other layers, including the cases of neighbourhood, societal and institutional levels. In addition, the relatively small sample size of the narrower age range of 6–8 months and the complex survey design may have affected power and the ability to detect a significant association such that further associations may have been missed.

## 5 CONCLUSION AND RECOMMENDATIONS

The low proportion of caregivers introducing solid, semisolid and soft foods between 6 and 8 months across survey rounds partly reflects the household structure supporting existing feeding practices. In large households, the primary caregivers do not get the support that enables her to introduce complementary foods in a timely manner with the time and effort given to others, a possible impediment. The presence of nonrelatives or other extended members changes the household structure, increasing the risk of early or late introduction of complementary foods. However, women from nuclear family households or smaller households with extended families with diversity in age and educational status were more likely to introduce complementary foods in a timely way.

In conclusion, the household structure and composition might influence the introduction of solid, semisolid and soft foods by influencing the availability and access of intrahousehold feeding and caregiving resources to the primary caregivers. The higher intrahousehold dependence of nuclear family households facilitates the appropriate introduction of complementary foods; thus interventions empowering each household member help to engage each in caregiving and enhance the quality of support to the primary caregiver. Also, feeding interventions might consider diverse roles and experiences associated with kinship status, noting that the positive influence is limited primarily to the nuclear family members. Further attention and action might be needed to address the negative influence of expanded and unrelated household members. Furthermore, national infant feeding programmes would likely benefit from considering household demographic diversity, including age and educational status. Finally, infant feeding intervention strategies must acknowledge variations in the sociocultural context that operate across Ethiopia.

## AUTHOR CONTRIBUTIONS

Asnake Ararsa Irenso conceived the research idea. Rachel Laws, Karen J. Campbell, Dan Chamberlain, Miaobing  Zheng and Asnake Ararsa Irenso developed the entire proposal, data processing and analysis. Asnake Ararsa Irenso drafted the manuscript. Rachel Laws, Karen J. Campbell, Miaobing  Zheng and Dan Chamberlain have contributed to the final manuscript by providing critical feedback on the results and their interpretation.

## CONFLICT OF INTEREST

The authors declare no conflict of interest.

## Data Availability

The data that support the findings of this study are available from the Demographic and Health Survey upon request.
